# Supplementary material for: Ripply suppresses Tbx6 to induce dynamic-to-static conversion in somite segmentation
Source: Nat Commun. 2023 Apr 13;14:2115. doi: 10.1038/s41467-023-37745-w (PMC10102234; doi:10.1038/s41467-023-37745-w)
Supplement: Supplementary file 1 — Supplementary Information [file 41467_2023_37745_MOESM1_ESM.pdf]

Supplementary information

Ripply suppresses Tbx6 to induce dynamic-to-static conversion in somite segmentation

Taijiro Yabe[1,2,3,*], Koichiro Uriu[4,*] & Shinji Takada[1,2,3,*]

Supplementary Fig. 1-22
Supplementary Table 1-2

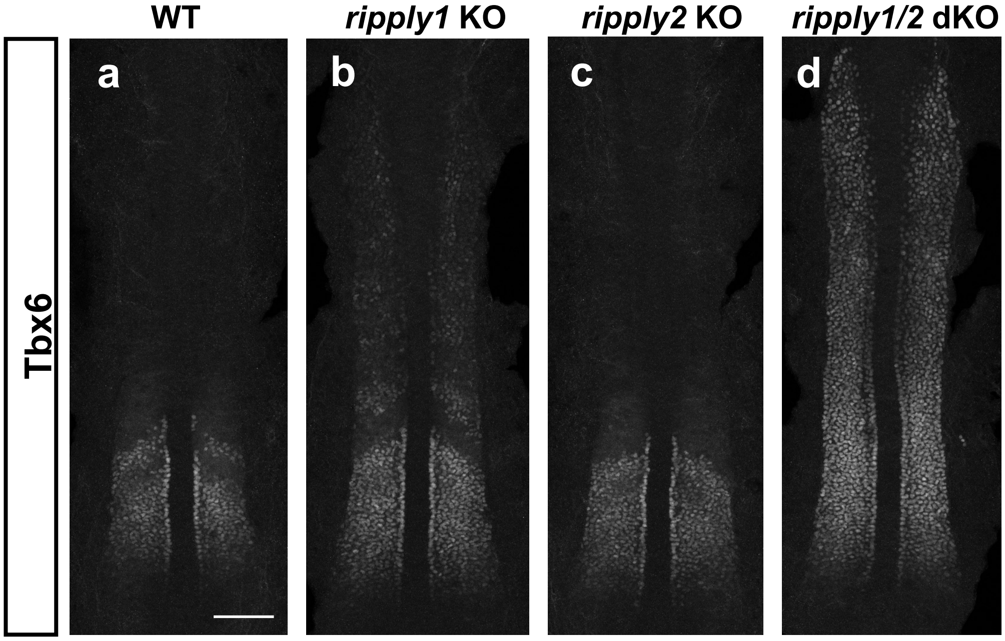

Supplementary Fig. 1. Partially redundant roles of *ripply1* and *ripply2* in removal of Tbx6 protein in the anterior paraxial mesoderm.

(a-d) Embryos were fixed at the 6-8-somite stage and Tbx6 expression was examined by immunohistochemical analysis. Embryos were flat-mounted after removal of yolk with heads toward the upper side. In wild-type (a; n=4) or *ripply2* homozygous embryos (c; n=3), the anterior border of the Tbx6 protein domain was evident as indicated by arrowheads. In the *ripply1* double mutant, although Tbx6 removal occurred in the anterior PSM, Tbx6 protein expression was restored in some cells of anterior paraxial mesoderm (b; n=3). In contrast, in ripply1 and ripply2 double-homozygous mutant embryos, Tbx6 protein was uniformly expressed in the paraxial mesoderm anterior to the anterior PSM (d; n=3). Scale bar indicates 100 µm.

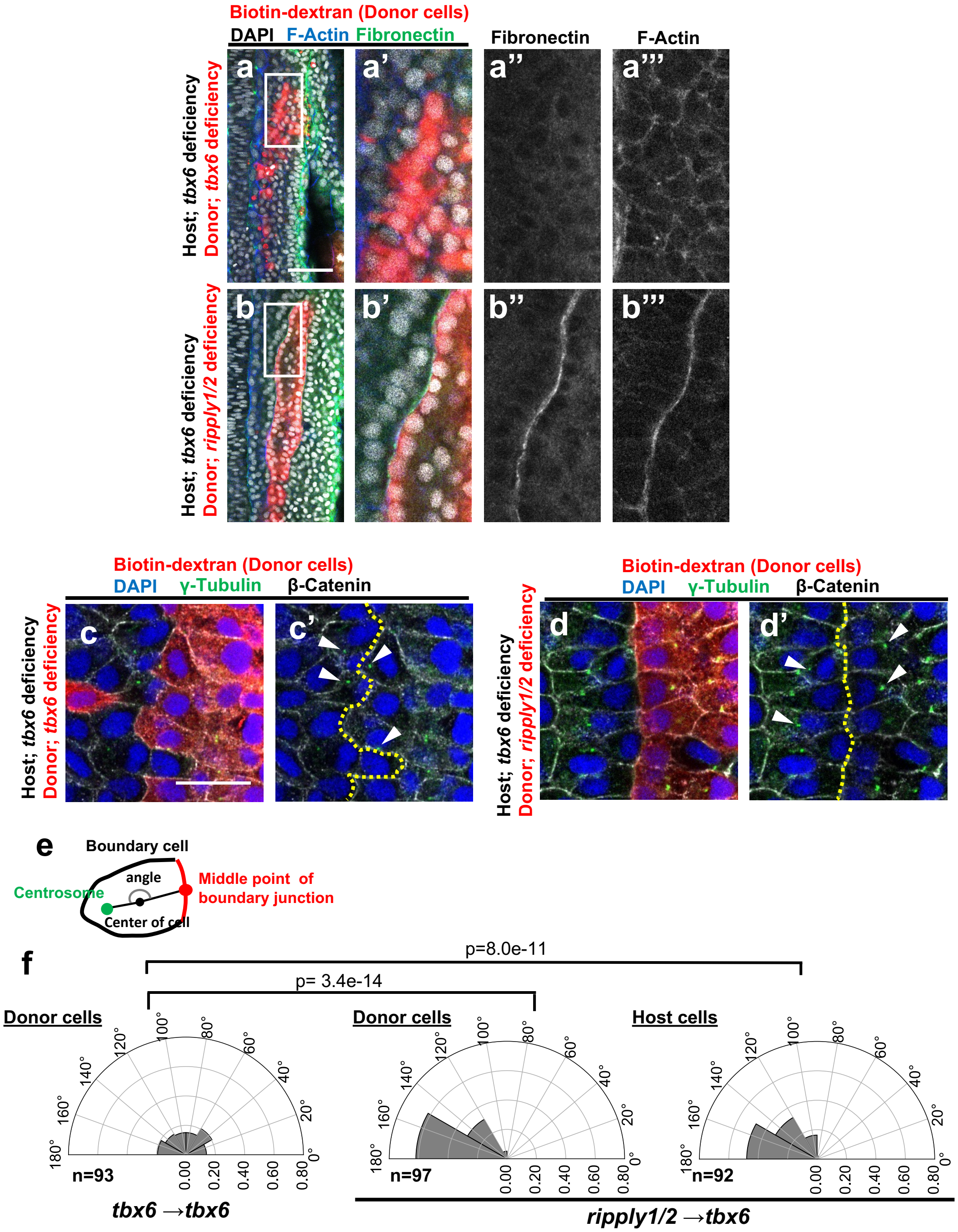

Supplementary Fig. 2. Accumulation of F-Actin at the edge of the Tbx6-expressing cell mass

(a,b) IHC staining of fibronectin (green) and phalloidin (blue) in *tbx6*-deficient embryos with transplanted *tbx6*-deficient donor cells(a; n=20) and *ripply1/2*-deficient donor cells (b; n=15). Donor cells were detected with rhodamine-labeled streptavidin (red). High-magnification images of the area

indicated by white boxes in c and d are shown on the right. Scale bar indicates 50 µm. (c, d) IHC staining of γ-Tubulin (green) and β-Catenin (white) in *tbx6*-deficient embryos with transplanted *tbx6*-deficient donor cells(c; n=14) and *ripply1/2*-deficient donor cells (d; n=15). Donor cells were detected with rhodamine-labeled streptavidin (red). White arrowheads indicate the centrosomes of boundary cells. Scale bar indicates 20 µm. (e) Schematic representation of the quantification of the position of the centrosome in boundary cell. (f) A statistical summary of experiments c and d. The angle of the centrosome relative to the middle position of the boundary junction in the boundary cell was plotted. The area of each bin represents the frequency. Differences were evaluated by pairwise comparisons using the two-sided Wilcoxon rank sum test adjusted for multiple comparison with Bonferroni's method.

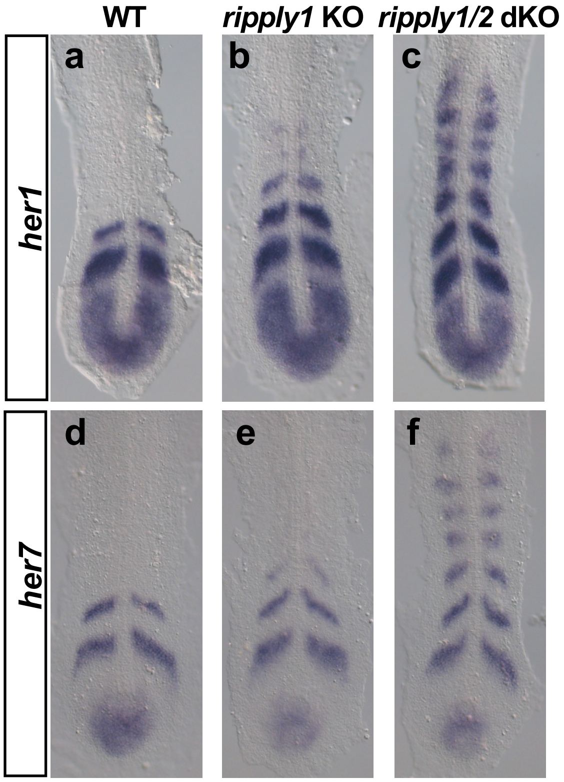

Supplementary Fig. 3. Anterior expansion of segmentation clock gene expression in *ripply1/2* dKO embryos.

(a-f) Expression of *her1* mRNA (a-c) and *her7* mRNA (d-f) at the 6-somite-stage of WT (a; n=8, d; n=11), *ripply1*$^{kt1032/kt1032}$ (b; n=7, e; n=6) and *ripply1*$^{kt1032/kt1032}$*; ripply2*$^{kt1034/kt1034}$ embryos (c; n=3, f; n=6). Embryos were flat-mounted after removal of yolk with heads toward the upper side.

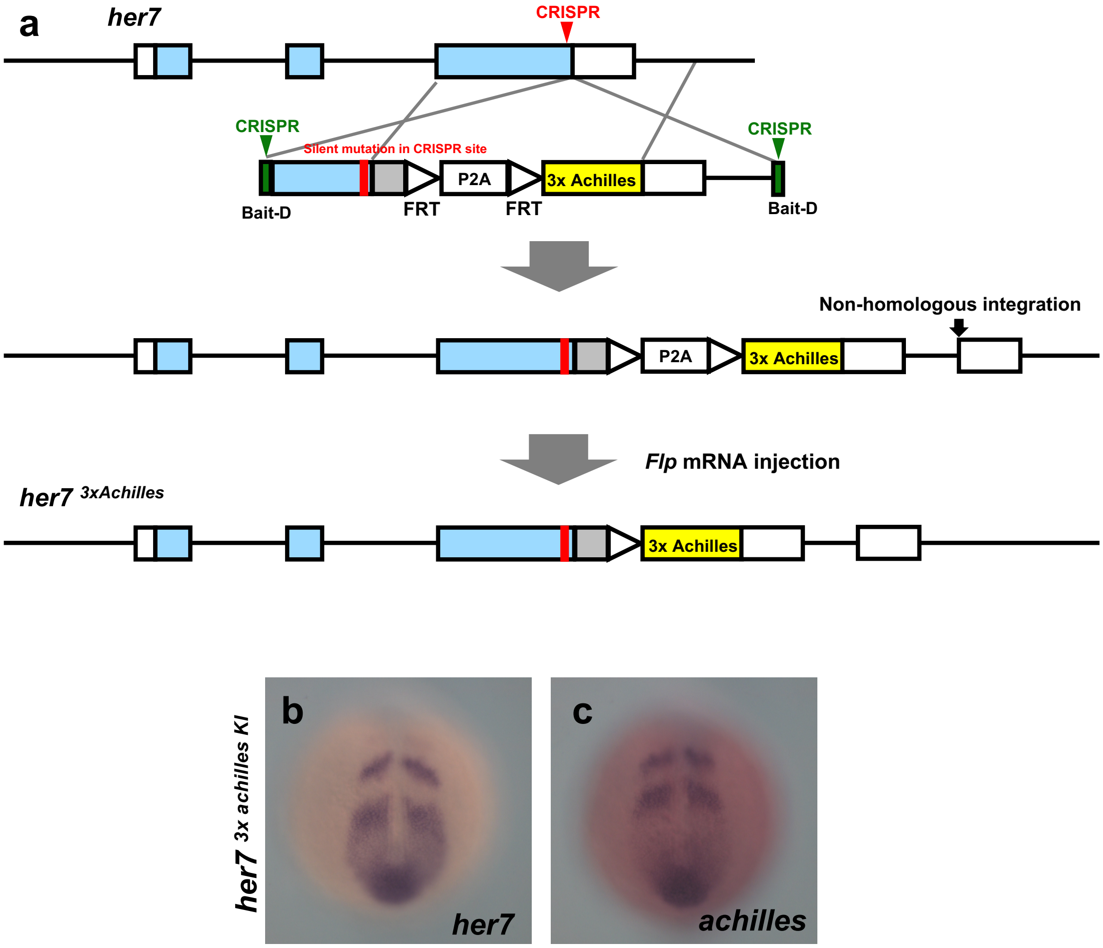

Supplementary Fig. 4. Generation of *her7*^{3xAchilles} KI fish

Schematic illustration of knock-in strategy (a). A targeting vector containing GS-linker (indicated by gray box), FRT-p2a-FRT cassette and tandemly connected three Achilles cDNAs flanked by a 500-bp homology arm with a silent mutation in the sgRNA recognition sequence were injected into early one-cell embryos with Cas9 mRNA and sgRNAs. KI fish were screened using the fluorescent signal of 3x Achilles which is separated from Her7 by the effects of P2A peptide. After screening, the p2a cassette was removed by *flp* mRNA injection. Although the 3' UTR was duplicated in the *her7*^{3xachilles} allele, which is probably caused by an unexpected non-homologous endo-joining, *her7-achilles* mRNA was expressed with a striped pattern in *her7*^{3xachilles} heterozygous embryos (b, c), suggesting that knock-in of 3x *achilles* and duplicated 3'UTR do not affect oscillatory expression of *her7*.

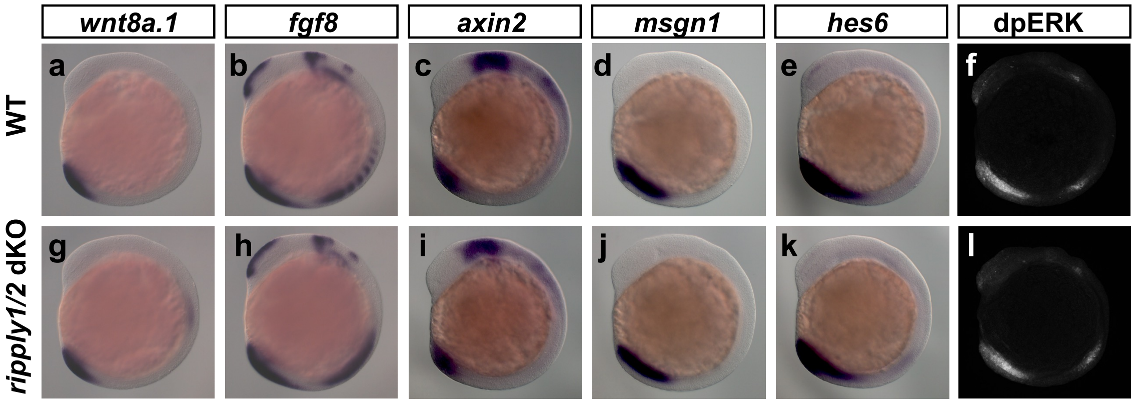

Supplementary Fig. 5. Intact Wnt and Fgf signal activity gradient in the PSM of *ripply1/2* double mutant embryo.

(a-l) Expression of *wnt8a.1* (a; n=11, g; n=10), *fgf8* (b; n=10, h; n=10), *axin2* (c; n=9, I; n=6), *msgn1* (d; n=8, j; n=5), *hes6*(e; n=9, k; n=7) and dpERK (f; n=3, l; n=2) in WT (a-f) and *ripply1*$^{kt1032/kt1032}$; *ripply2*$^{kt1034/kt1034}$ embryos (g-h) at the 7-somite- stage with dorsal toward the right.

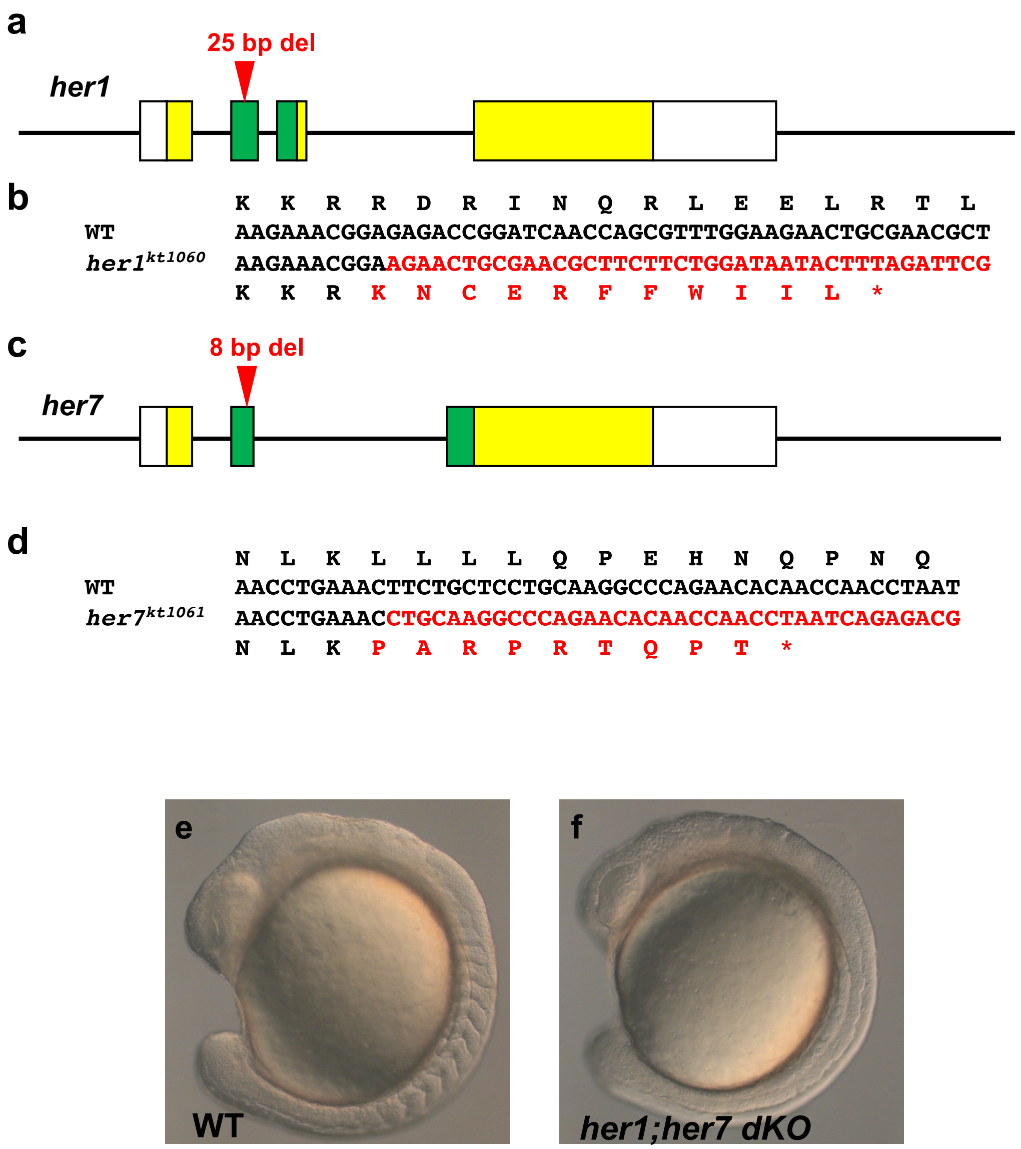

Supplementary Fig. 6. Generation of *her1* and *her7* mutants

Schematic illustration of *her1* and *her7* genes (a, c). Colored boxes indicate protein coding regions. Green boxes indicate regions encoding bHLH domains, essential for DNA binding. DNA and protein sequences of wild-type and mutants are shown in b and d. A premature stop codon generated by a frame-shift is identified by an asterisk. $her1^{kt1060}$ and $her7^{kt1061}$ double-homozygous embryos exhibited severely impaired somite boundary formation, as previously reported in other alleles (e, f).

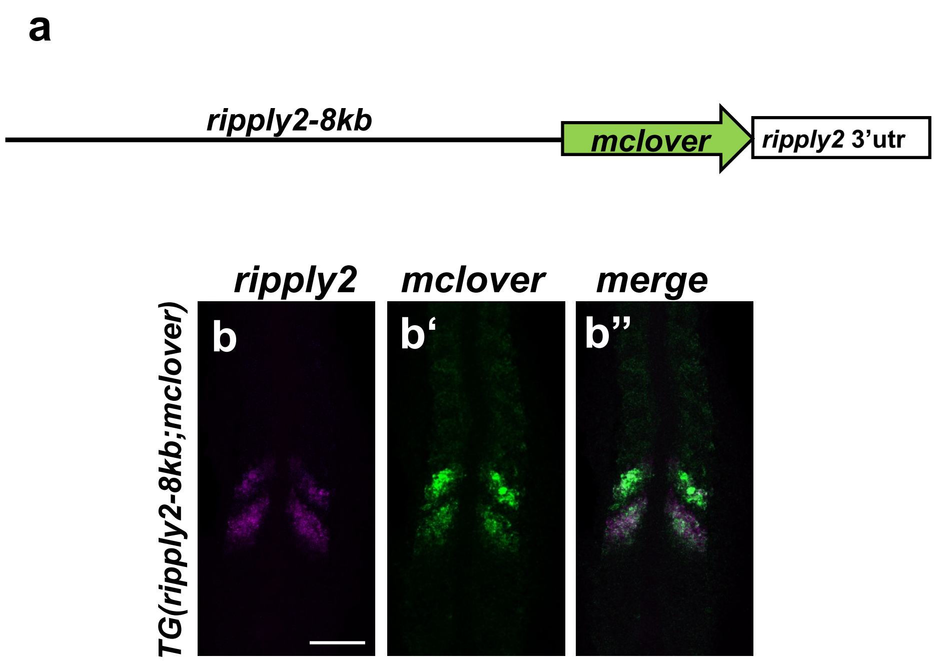

Supplementary Fig. 7. Recapture of endogenous *ripply2* expression by *ripply2-8kb* promoter

(a)Schematic illustration of a construct for transgenesis. (b)Expression of *ripply2* (magenta) and *mclover* (green) mRNA in *TG(ripply2-8kb;mclover)* transgenic embryos. Scale bar indicates 100 µm.

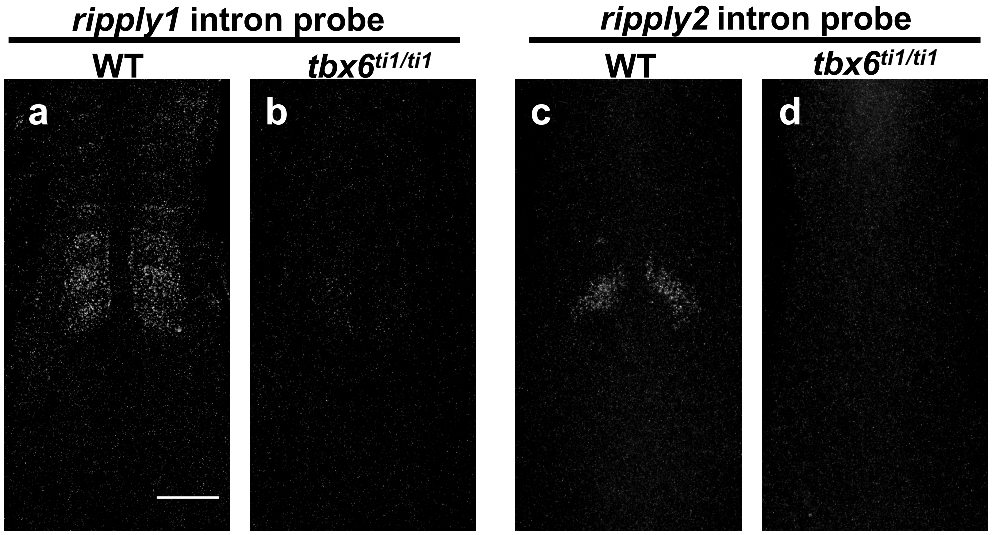

Supplementary Fig. 8. Regulation of *ripply1* and *ripply2* expression by Tbx6

Expression of *ripply1* (a; n=6, b; n=6) and *ripply2* (c; n=6, d; n=5) nascent mRNA was examined in wild-type (a, c) and $tbx6^{ti1}$ homozygous embryos (b, d) by fluorescent *in situ* hybridization. Embryos were fixed at the 6-7-somite stage and flat-mounted after removal of yolk with heads toward the upper side. Transcription of *ripply1* and *ripply2* was severely decreased by disruption of Tbx6 function. Scale bar indicates 100 µm.

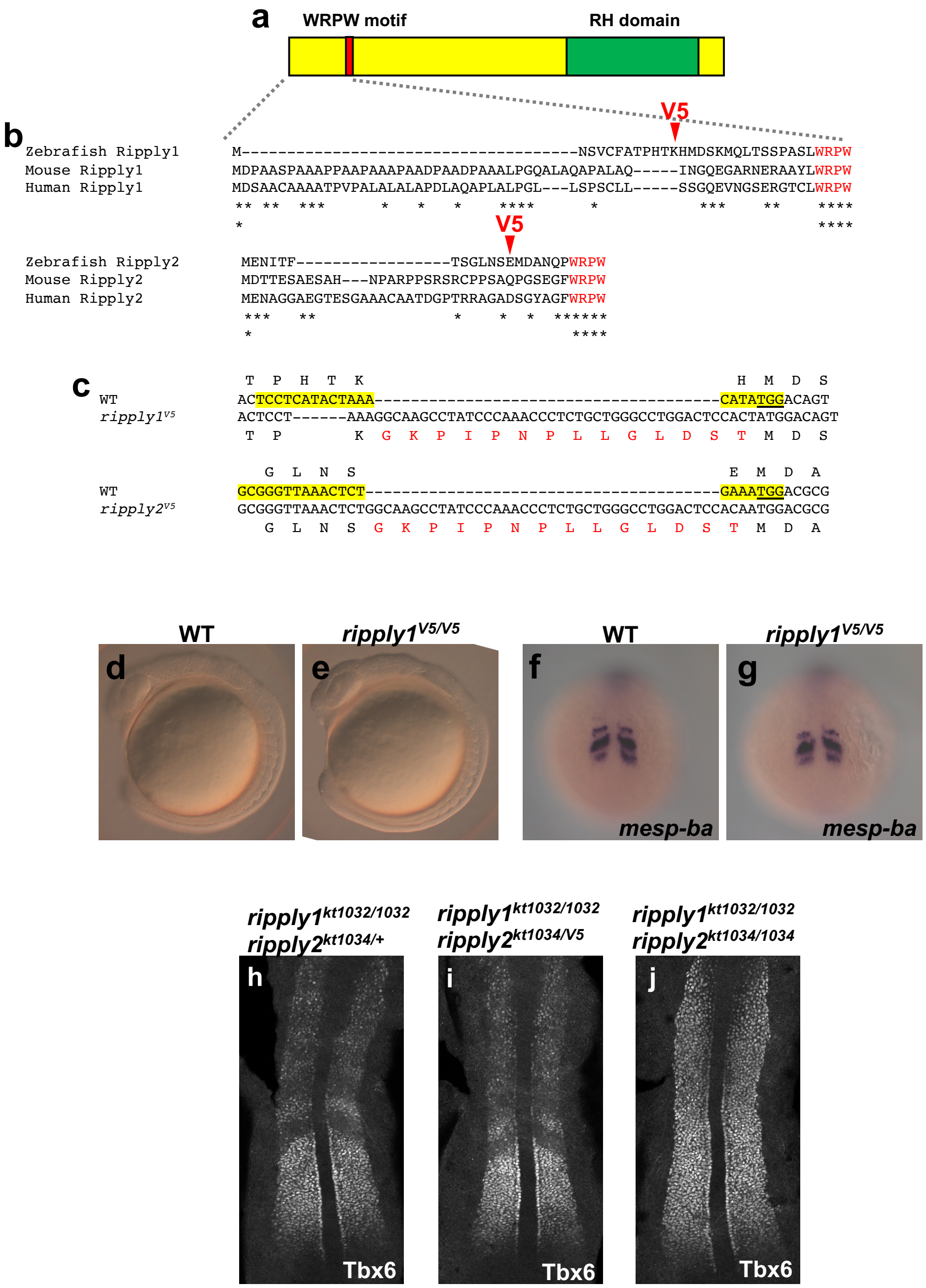

Supplementary Fig. 9. Tagging of endogenous Ripply proteins by ssODN-mediated genome editing

(a) Schematic image of Ripply protein. Red and green boxes indicate WRPW motifs and Ripply-homology domains (RH domains), which are essential for ripply function. (b) Protein alignment of

the N-terminal non-conserved region. An arrowhead indicates the position of V5 tag insertion. (c) Nucleic acid sequences neighboring the V5 tag insertion site in WT and KI fish. In the ripply1v5 allele, an unexpected 6-base insertion was introduced upstream of v5 tag insertion without induction of a frameshift. (d-g) Validation of *ripply1* activity in *ripply1*^{v5} knock-in embryos. *ripply1v5* homozygous embryos do not show any defects in somite boundary formation (d; n=3, e; n=5) or normal *mespba* expression (f; n=2, g; n=3), whereas severe defects in somite boundary formation and anterior expansion of *mespba* expression were reported in ripply1 deficient embryos, suggesting v5-tagged Ripply1 protein still possesses enough activity to control normal somitogenesis. (h-j) Validation of *ripply2* activity in *ripply2*^{v5} knock-in embryos. The phenotype of *ripply1*$^{-/-}$*; ripply2*$^{+/-}$ embryos was milder than that of *ripply1; ripply2* double-homozygous embryos in regard to anterior expansion of Tbx6 protein in paraxial mesoderm (h; n=3, j; n=3). The phenotype of *ripply1*$^{-/-}$*; ripply2*$^{v5/-}$ was indistinguishable from that of *ripply1*$^{-/-}$*; ripply2*$^{+/-}$ (i; n=2), suggesting that *ripply2*^{v5} possesses activity comparable to wild-type *ripply2* allele. Scale bar indicates 100 µm.

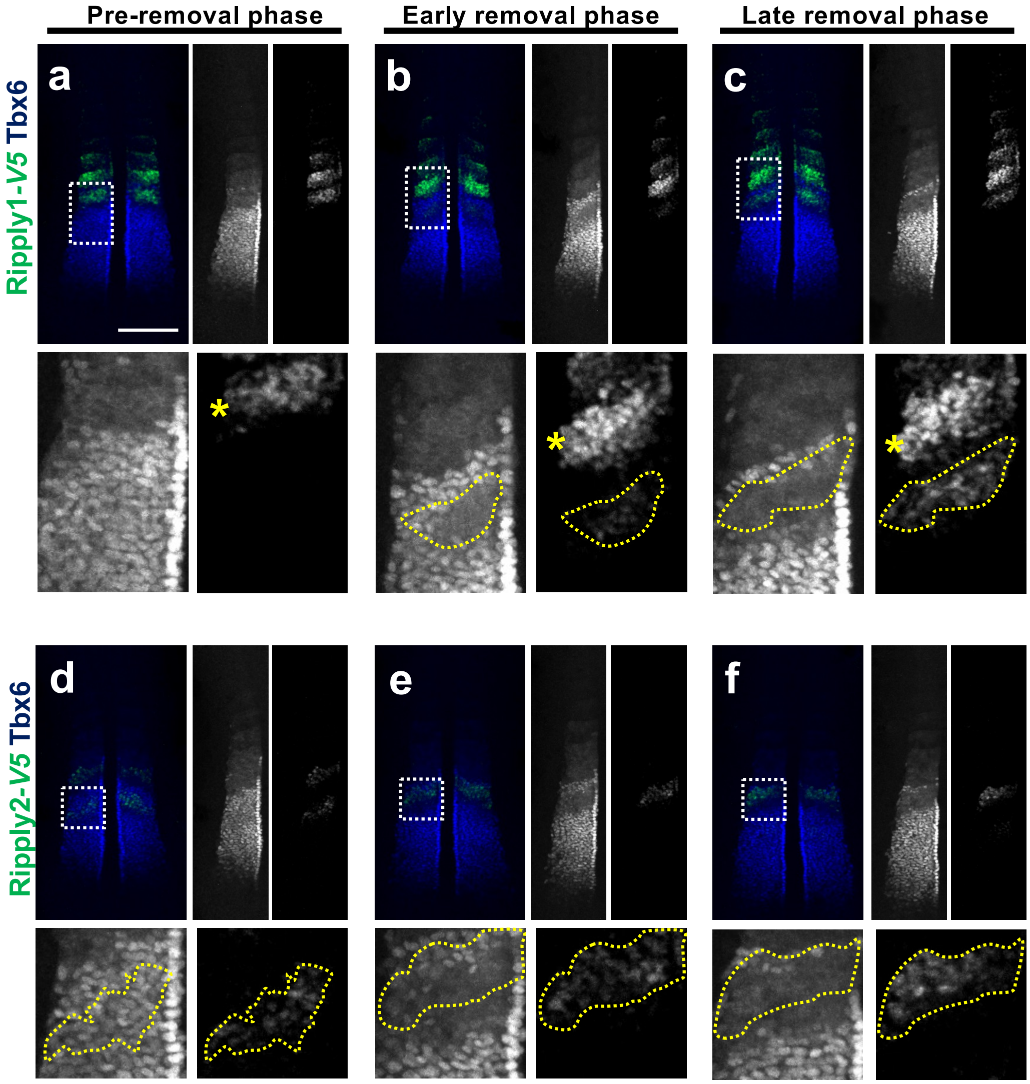

Supplementary Fig. 10. Dynamics of Ripply1/2 and Tbx6 protein during somitogenesis.

A series of fixed *ripply1*^{V5} heterozygous (a-c) and *ripply2*^{V5} homozygous (d-e) embryos at the 6-7-somite stage. Tbx6 protein (blue) and Ripply1 or Ripply2 protein (green) were detected immunohistochemically (a; n=9, b; n=8, c; n=8, d; n=10, e; n=6, f; n=9). Individual channel images are shown at the right. Magnified images of individual channels surrounded by white dotted squares are shown below. Areas surrounded by yellow dotted lines indicate the domain of most posterior striped of Ripply-V5-expressing domain. Yellow asterisks indicate the anterior Ripply1-V5-expressing domain. Timing of somitogenesis was estimated according to expression of Tbx6 protein at the anterior PSM. Scale bar indicates 100 µm.

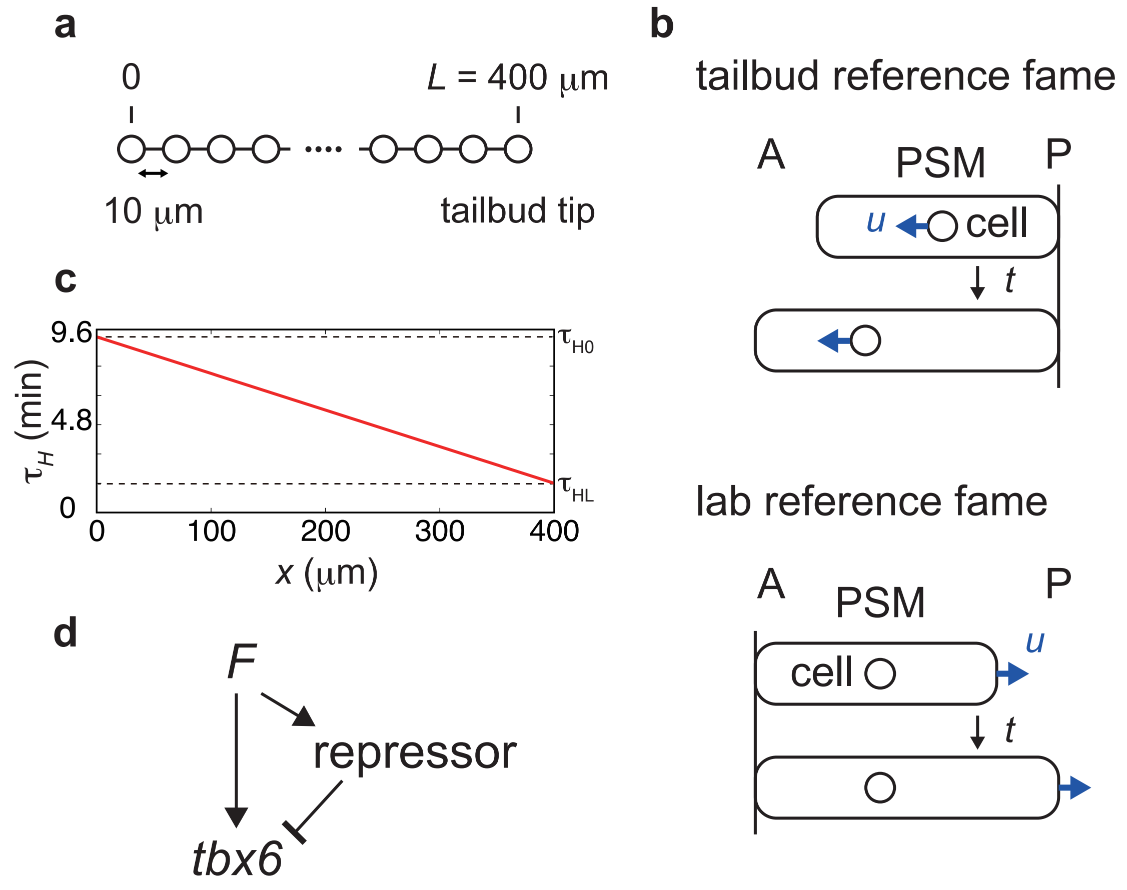

Supplementary Fig. 11. Scheme of the mathematical model.

(a) A one-dimensional array of cells represents the PSM. (b) Tailbud and lab reference frames. (c Spatial dependence of translational time delay of Her protein. (d) Assumed incoherent feedforward loop that regulates Tbx6 production.

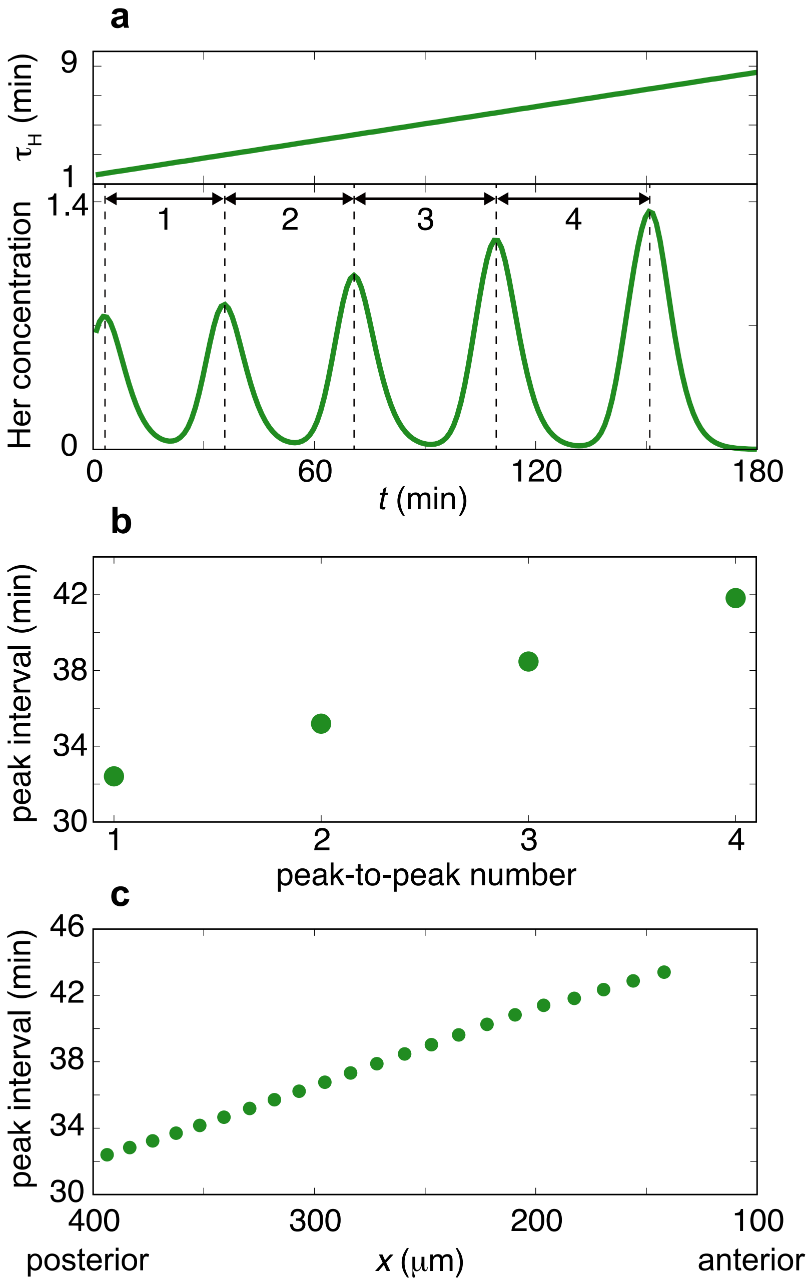

Supplementary Fig. 12. Spatial and temporal changes of Her oscillation.

(a) Time evolution of (top) translation time delay τ_H and (bottom) concentration of Her protein in a single cell in simulations. Peak-to-peak intervals are indicated by arrows and each interval is assigned a number as indicated. (b) Lengthening of peak intervals at each oscillation cycle. Nnumbers in the horizontal axis correspond to those in (a). (c) Spatial dependence of peak intervals. Because cell

positions relative to the tailbud change from posterior to anterior, the horizontal axis is inverted as larger values on the left.

(a)

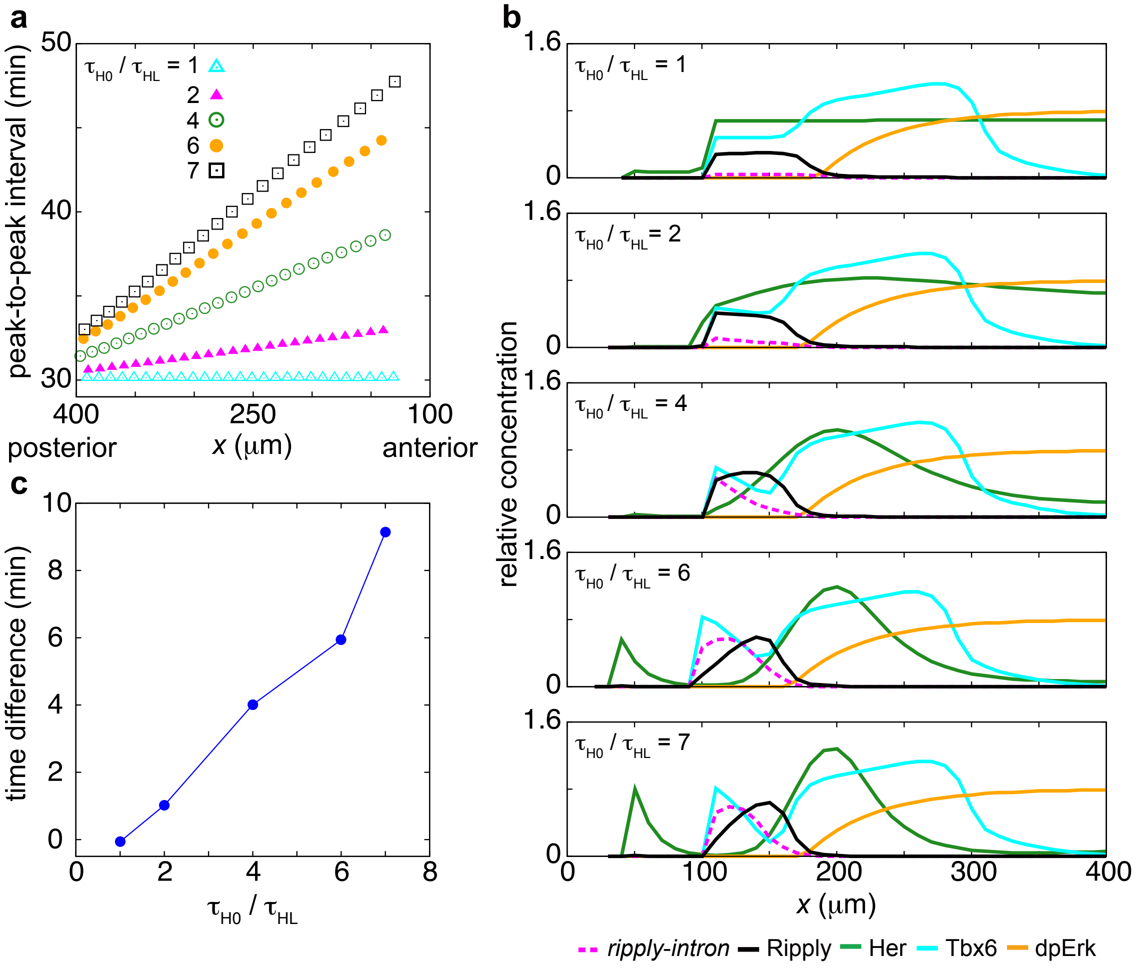

(b)

Supplementary Fig. 13. Dependence of the Tbx6 removal pattern on Her waves.

(a) Spatial dependence of peak intervals of Her oscillation with different ratios of translation delays. $\tau_{HL} = 1.6$ min is the translation delay at the right end of the lattice (tailbud tip). τ_{H0} is the translation delay at the left end of the lattice (400 μm anterior to the tailbud). Because cell positions relative to the tailbud change from posterior to anterior, the horizontal axis is inverted with larger values on the left. (b) Snapshots of *ripply-intron* mRNA, and those of Ripply, Her, Tbx6, and dpErk proteins in simulations with different values of τ_{H0}/τ_{HL}. Anterior is on the left. As the ratio of time delays increases, Her oscillation slows and the wavelength of Her expression shortens, causing a large time difference of Tbx6 removal between cells near the dpErk edge and those near the anterior Tbx6

boundary. (c) Dependence of the time difference of Tbx6 removal on the ratio of translation delays τ_{H0}/τ_{HL}. We measured the time at which Tbx6 levels crossed 0.5 by Ripply-induced degradation in 6 cells included in a single prospective somite. We then computed the time difference between the most anterior and the second most posterior cells among the 6 cells.

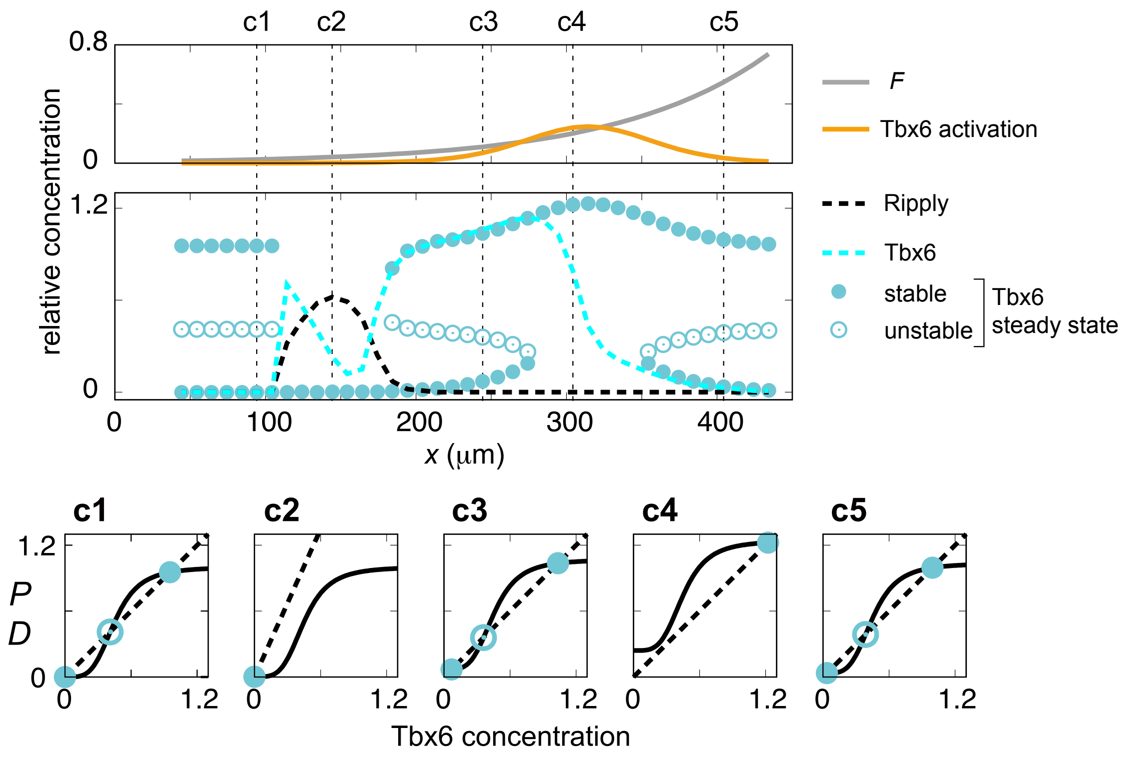

Supplementary Fig. 14. Steady states of Tbx6 protein across the entire PSM.

This figure is the full version of Fig. 7c in the main text showing the entire PSM region. (Top) Fgf/Erk signaling F and Tbx6 activation by the incoherent feedforward loop are plotted as a function of spatial position. (bottom) Tbx6 steady states as a function of spatial position. Cyan and black broken lines indicate snapshots of Tbx6 and Ripply levels, respectively, obtained by simulation. Anterior is on the left. (c1-c5) Dependence of the production speed P of Tbx6 (solid line) and its degradation speed D (broken line) on Tbx6 protein levels at each spatial position indicated by numbers (1–5) in the top panel. See the Methods for the definition of P and D. Circles indicate the intersection of these two lines. Filled and open circles indicate stable and unstable Tbx6 steady states, respectively.

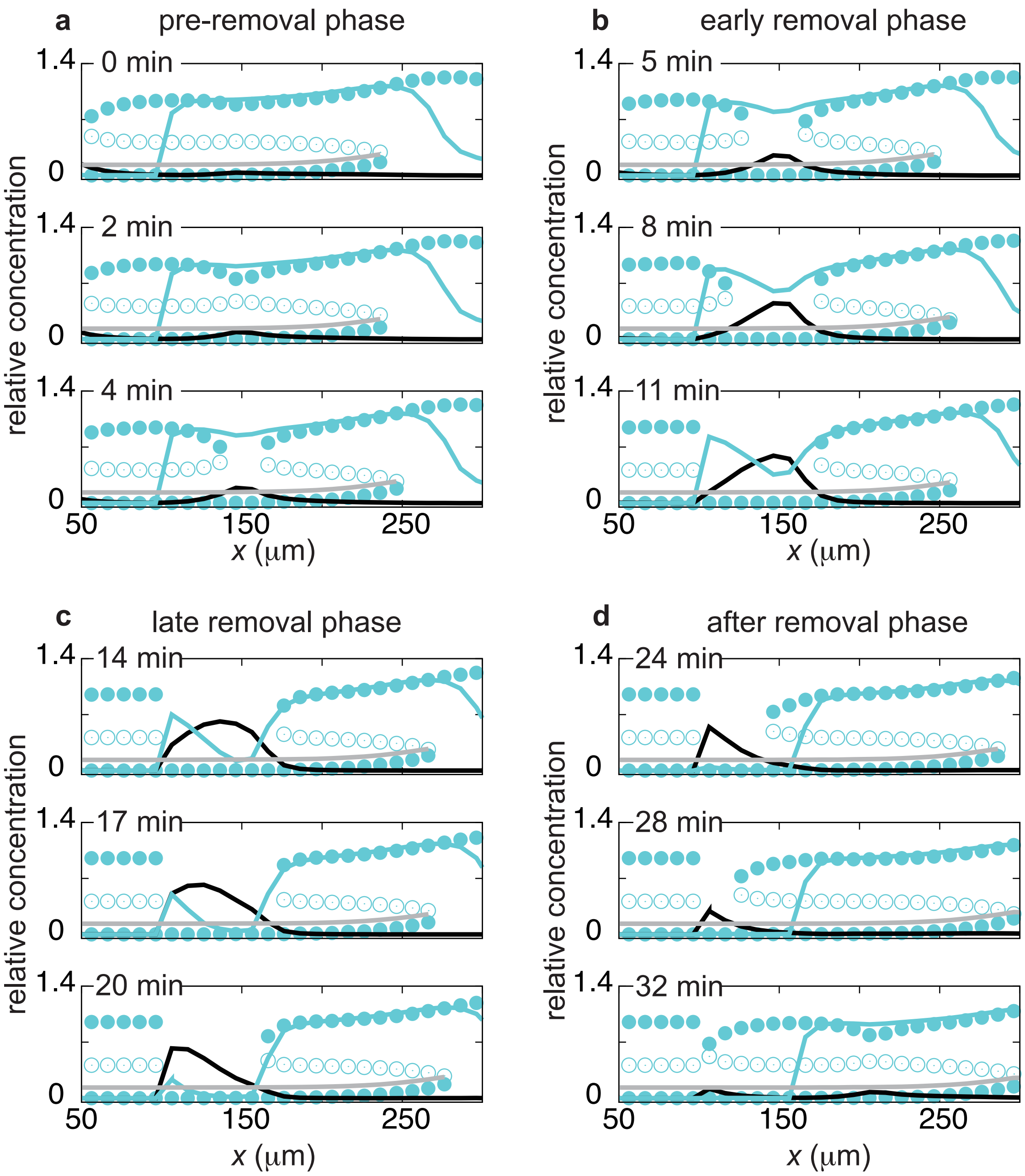

Supplementary Fig. 15. Time evolution of Tbx6 steady states in the anterior PSM.

(a)-(d) Snapshots of spatial patterns of Tbx6 protein (cyan lines), Ripply protein (black lines), stable (filled circles) and unstable (open circles) steady states of Tbx6 protein. Gray lines indicate Ripply protein levels at which a saddle-node (SN) bifurcation occurs. If Ripply protein levels exceed the gray line, the higher stable steady state and the unstable steady state collide and disappear, while the lower

Tbx6 steady state remains stable. (a) In pre-removal phase, Ripply protein levels start increasing. As Ripply levels become larger than the gray line, SN bifurcation occurs (4 min). (b) In early removal phase, a sequence of SN bifurcation occurs across cells. (c) In late removal phase, Tbx6 protein is degraded by Ripply protein, converging to the lower steady states. Convergence occurs from posterior to anterior. (d) After removal of Tbx6, Ripply protein is also removed by degradation, recovering bistability in a prospective somite region. Tbx6 protein levels remain low.

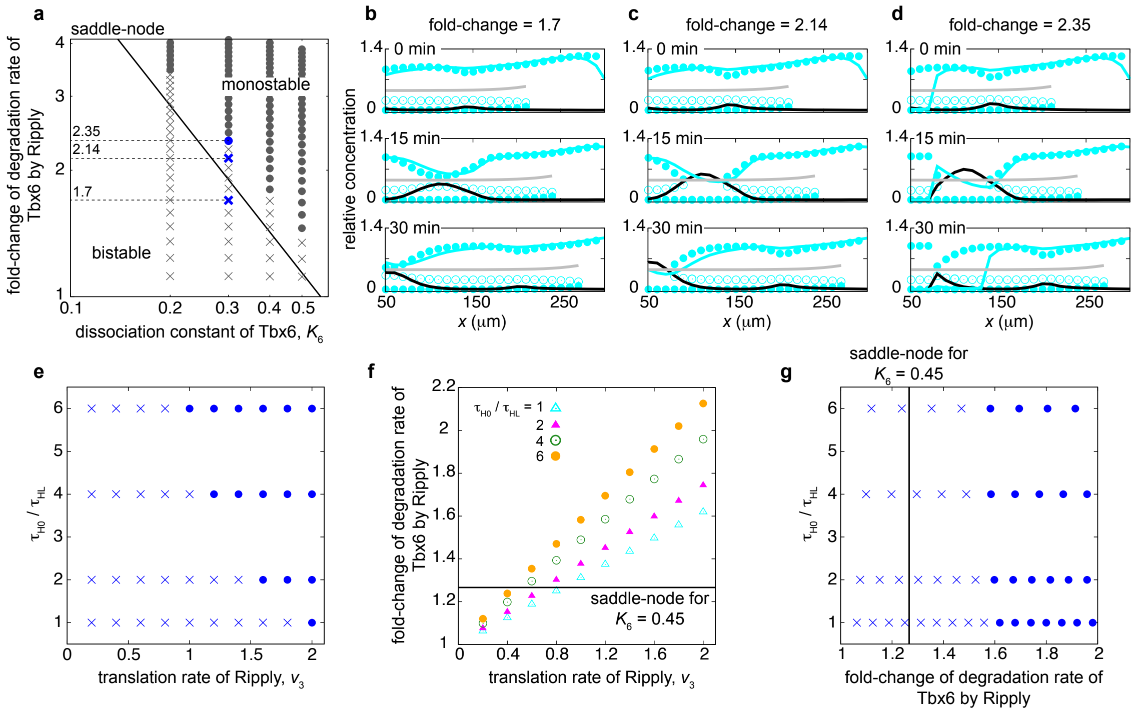

Supplementary Fig. 16. Change of the Tbx6 degradation rate by Ripply is required for somite boundary formation.

(a) Phase diagram of Tbx6 boundary formation (same as Fig. 7d in the main text). Blue symbols indicate parameter combinations of which results are shown in (b)-(d). To change the Tbx6 degradation rate, Ripply translation rate (v_3 in Eq. (4) in Methods) was varied. (b)-(d) Snapshots of spatial patterns of Tbx6 protein (cyan lines), Ripply protein (black lines), stable (filled circles) and unstable (open circles) steady states of Tbx6 protein with different fold-change values of Tbx6 degradation rate by Ripply. Gray lines indicate Ripply protein levels at which a saddle-node (SN) bifurcation occurs. (b) Ripply protein levels do not exceed the threshold for SN bifurcation, failing Tbx6 boundary formation. (c) SN bifurcation occurs, but the degradation rate of Tbx6 is not high enough to degrade Tbx6 to lower steady states while Ripply is expressed. (d) Ripply protein levels are large enough to cause SN bifurcations and to degrade Tbx6 to lower steady states. (e)-(g) Slowing of Her oscillation in the anterior PSM facilitates Tbx6 boundary formation by increasing Tbx6 degradation rate. In (e)-(g), $K_6 = 0.45$. (e) Phase diagram of Tbx6 boundary formation with translation rate of Ripply v_3 as the horizontal axis and the ratio of translation delays of Her protein τ_{H0}/τ_{HL} as the vertical axis. A larger value in the vertical axis indicates slower oscillation of Her in

the anterior PSM. Circles indicate correct Tbx6 boundary formation. Crosses indicate failure in Tbx6 boundary formation until a wave of Ripply traveled the distance of one somite (6 cells). (f) Change of the Tbx6 degradation rate as a function of v_3 with different ratios of translation delays of Her protein. (g) Phase diagram of Tbx6 boundary formation with a change of the Tbx6 degradation rate by Ripply as the horizontal axis and the ratio of translation delays of Her protein τ_{H0}/τ_{HL} as the vertical axis. Symbols are the same as in (e). In (f) and (g), black lines indicate the change value required for a saddle-node bifurcation with $K_6 = 0.45$.

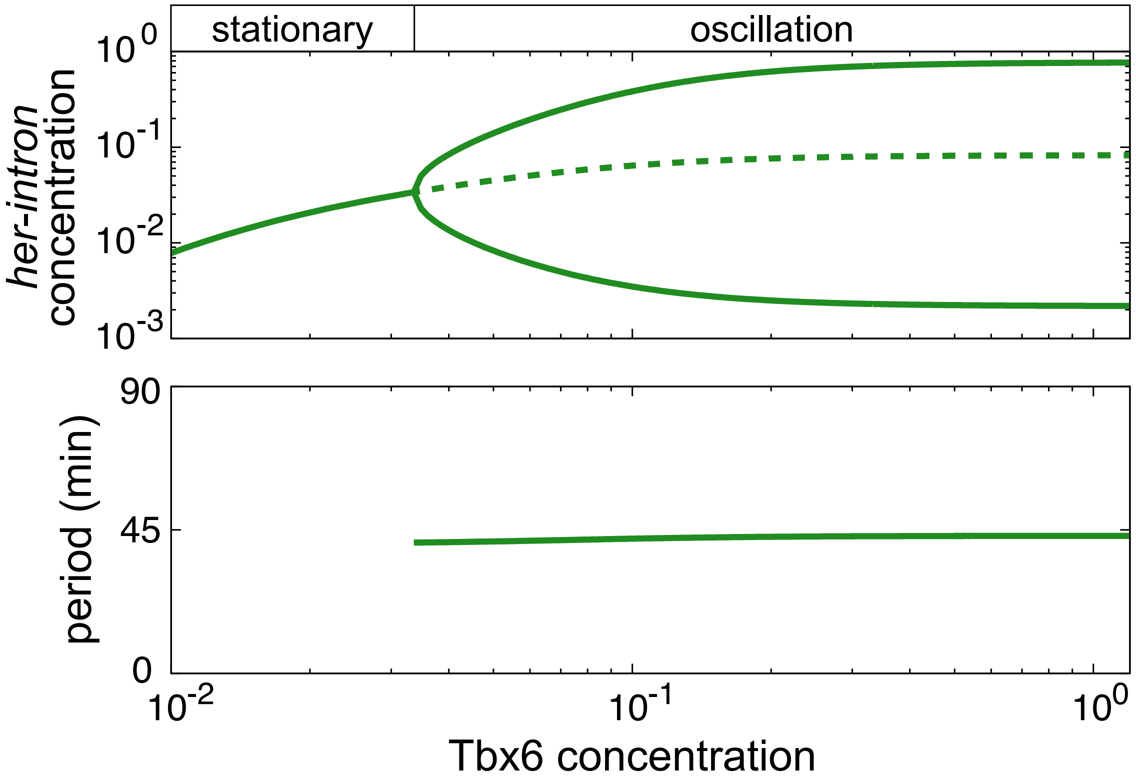

Supplementary Fig. 17. Dependence of *her* expression on Tbx6 protein levels.

(Top) Bifurcation diagram of *her-intron* mRNA levels. Oscillation of *her* expression stops via a Hopf bifurcation as Tbx6 protein levels decrease in the mathematical model. If *her* mRNA levels converge to a steady state, steady state values are plotted (solid line). If *her* mRNA levels oscillate, both the peak and trough of oscillation are plotted (solid lines) with an unstable steady state (broken line). (Bottom) Period of oscillation as a function of Tbx6 protein levels. Diagrams were obtained by numerical integration of delay differential equations for *her* mRNAs and Her protein with constant values of Tbx6 protein. See methods section for details.

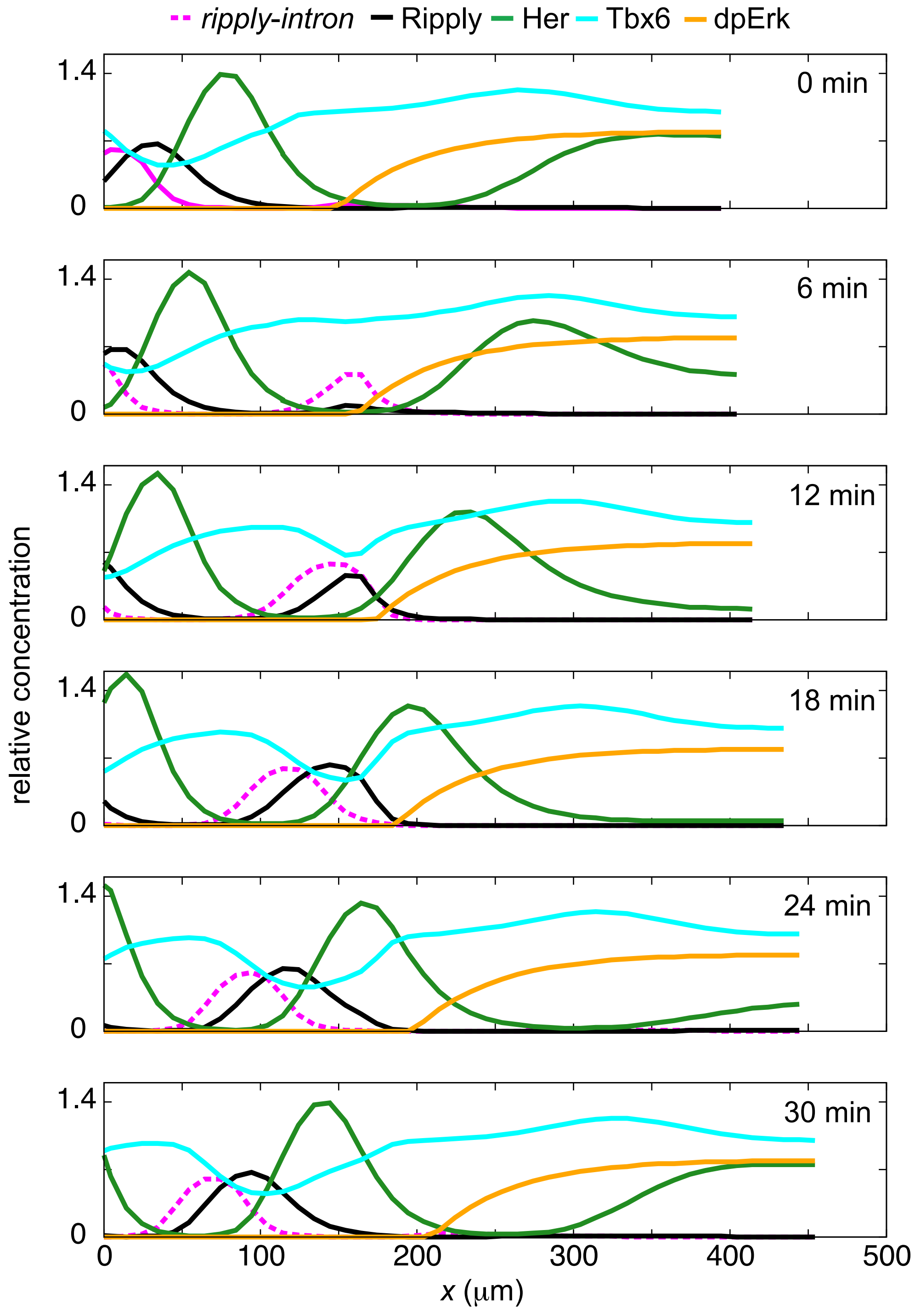

Supplementary Fig. 18. Failure in Tbx6 anterior border formation in the absence of Tbx6 auto-regulation.

Spatial patterns of Her protein, *ripply-intron* mRNA, Ripply protein, Tbx6 protein, and dpErk protein

are shown. Anterior is on the left. The positive feedback loop in the equation for Tbx6 protein levels was replaced with a constant value. See the Methods for details. All other parameter values are the same as listed in Supplementary Table 1.

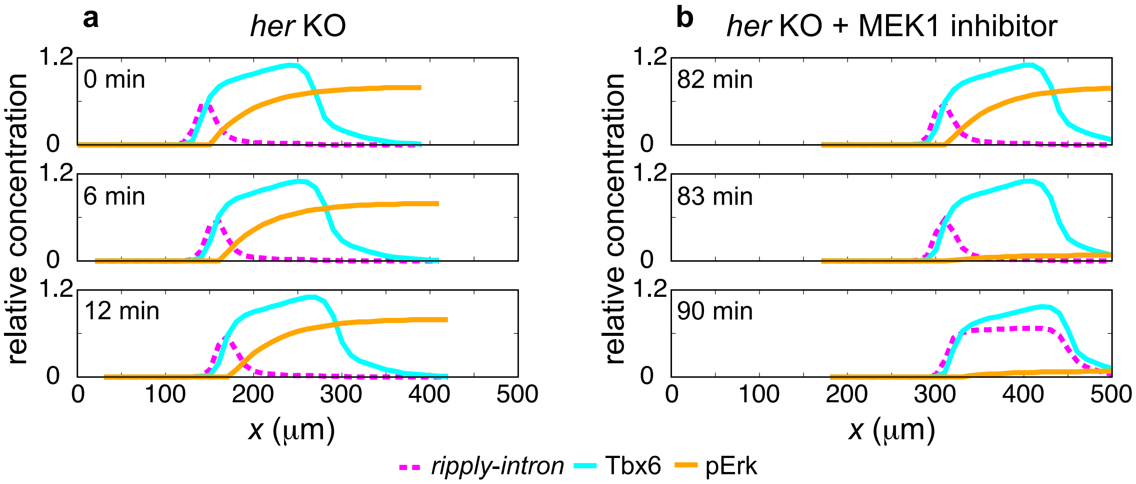

Supplementary Fig. 19. Simulation of *her* knock-out mutants. (a), (b) Snapshots of *ripply-intron* mRNA, Tbx6, and dpErk proteins in the absence of *her* transcription. At 82 min, dpErk levels are reduced by 10-fold in (b). The transcription term in the equation for *her-intron* mRNA is multiplied by zero. All other parameter values are the same as those listed in Supplementary Table 1.

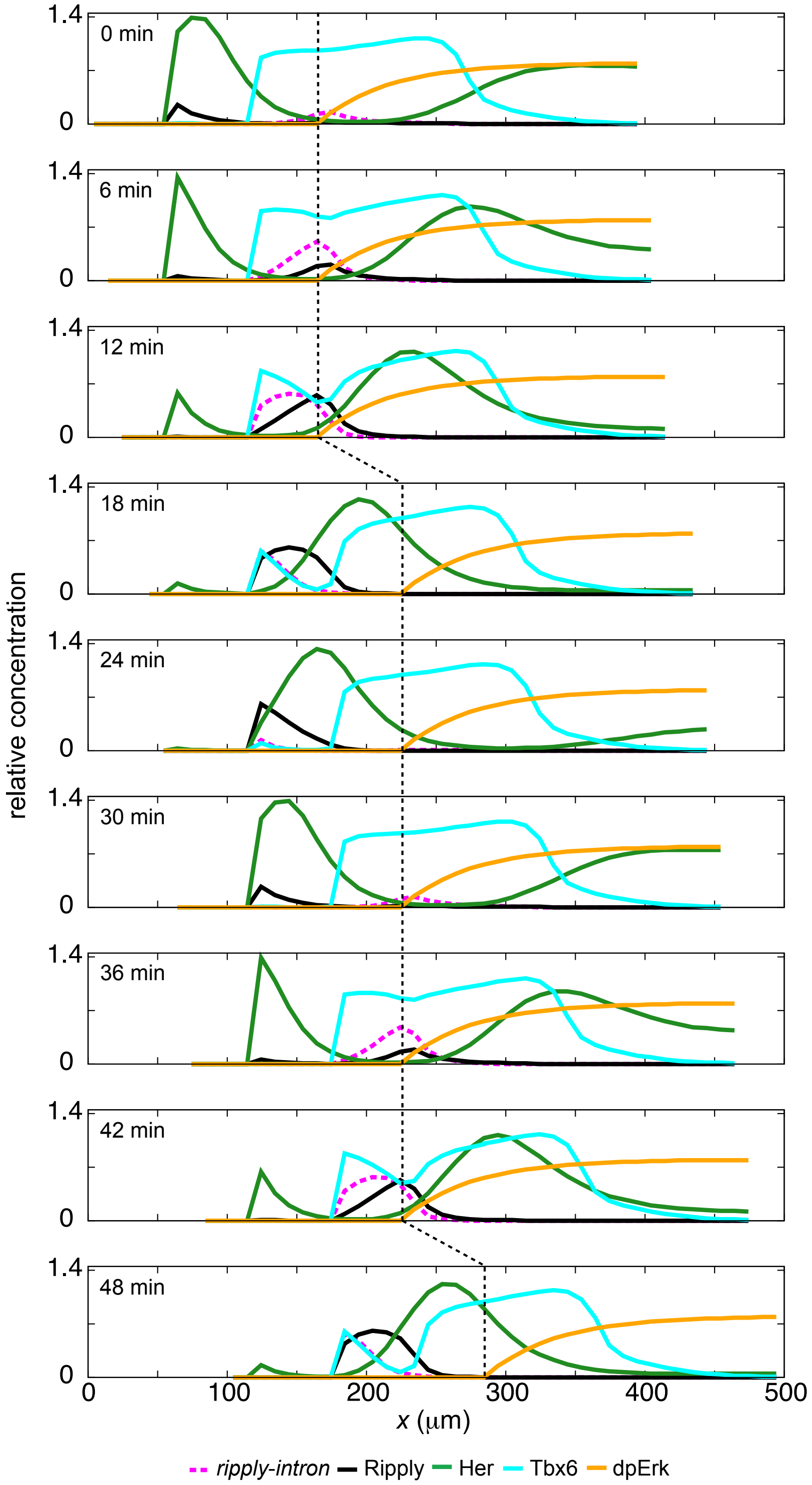

Supplementary Fig. 20. Periodic generation of Tbx6 anterior borders and clock arrest in the presence of a posterior stepwise shift of the dpErk gradient. Snapshots of *ripply-intron* mRNA,

Ripply, Her, Tbx6 and dpErk proteins are shown. Note the abrupt posterior shifts of the dpErk profile indicated by vertical broken lines. Parameter values are the same as those listed in Table S1. See the Methods for modeling of a stepwise shift of the dpErk gradient.

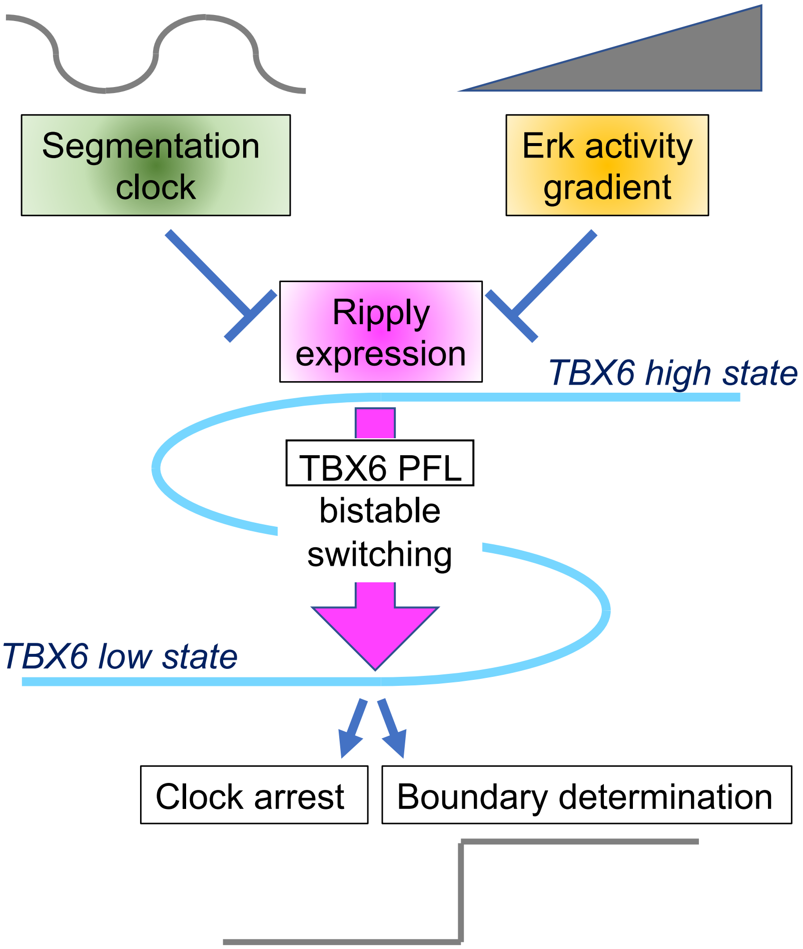

Supplementary Fig. 21. Schematic diagram of the mechanism underling dynamic-to-static conversion in zebrafish somitogenesis

Oscillatory expression of Her genes, the segmentation clock, cooperatively represses expression of ripply genes along with the Erk activity gradient, resulting in cyclic induction of Ripply expression. Once Ripply trigger a decrease in Tbx6 protein, the positive feedback loop (PFL) of Tbx6 should further decrease tbx6 expression and finally stabilize this expression at a low level. As a result, the position of the anterior border of the Tbx6 protein domain, which is corresponds to a future somite boundary, is stabilized and travelling of the Her oscillatory wave stops at this border.

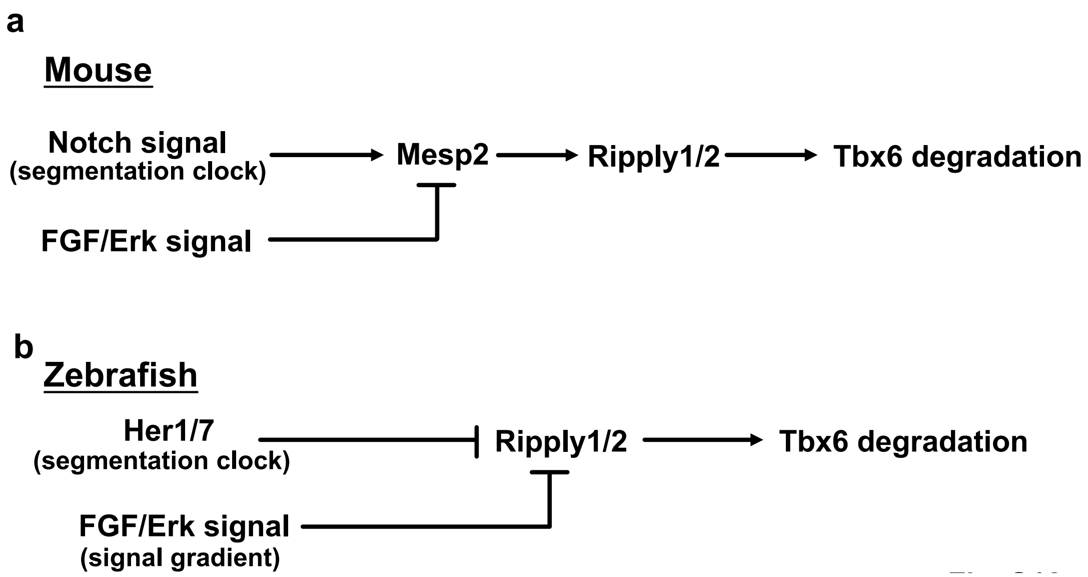

Supplementary Fig. 22. Diverse regulation of *ripply* expression in mice and zebrafish

Schematic illustration of regulation of Ripply in mice (a) and zebrafish (b). In mouse somitogenesis, Mesp2 is essential for expression of Ripply1 and Ripply2 in the anterior PSM (Ref.). Expression of Mesp2 is induced by Notch signaling, one of the major components of the mouse segmentation clock. Therefore, in mouse somitogenesis, the segmentation clock cyclically activates *Ripply* expression through the Notch-Mesp2 axis. In mouse somitogenesis, Fgf/Erk signaling also regulates Ripply expression through repression of Mesp2 expression, suggesting that the spatial-temporal information provided by the segmentation clock and signal gradient are integrated through regulation of *Mesp2* expression during mouse somitogenesis. On the other hand, the segmentation clock cyclically represses expression of *ripply* through functions of *hairy-related* genes to achieve periodicity of *ripply* expression in zebrafish somitogenesis. Unlike mice, in zebrafish, spatio-temporal information is integrated through regulation of *ripply* expression without the functions of Notch and Mesp.

Supplementary Table 1

Parameter values for wild type simulation

parameter	value	description
τ_h	5.9 min	transcriptional delay of *her* mRNA
τ_r	1.6 min	transcriptional delay of *ripply* mRNA
τ_{H0}	9.6 min	translational delay of Her protein at the anterior end of the array
τ_{HL}	1.6 min	translational delay of Her protein at the posterior end of the array
τ_R	1.6 min	translational delay of Ripply protein
n_1	2	Hill coefficient for *her* repression by Her protein
n_2	2	Hill coefficient for *her* activation by Tbx6 protein
n_3	2	Hill coefficient for *ripply* repression by Her protein
n_4	3	Hill coefficient for *ripply* repression by dpErk protein
n_5	2	Hill coefficient for *ripply* activation by Tbx6 protein
n_6	4	Hill coefficient for *tbx6* activation by Tbx6 protein
n_R	4	Hill coefficient for *tbx6* repression by Fgf/Erk
n_A	4	Hill coefficient for *tbx6* activation by Fgf/Erk
v_1	1.0	maximum transcription rate of *ripply*
v_2	3.85	translation rate of Her protein
v_3	2.2	translation rate of Ripply protein
μ_{sh}	1.16	splicing rate of *her* mRNA
μ_{sr}	1.16	splicing rate of *ripply* mRNA
μ_1	0.38 min^{-1}	degradation rate of *her* mRNA
μ_2	1.0	degradation rate of *ripply* mRNA
μ_3	0.19 min^{-1}	degradation rate of Tbx6 protein
μ_4	2.0	degradation rate of Her protein
μ_5	2.0	degradation rate of Ripply protein
K_1	0.06	threshold of Her protein for *her* repression
K_2	0.1	threshold of Tbx6 protein for *her* activation
K_3	0.06	threshold of Her protein for *ripply* repression
K_4	0.2	threshold of dpErk protein for *ripply* repression
K_5	0.45	threshold of Tbx6 protein for *ripply* activation
K_6	0.45	threshold of Tbx6 protein for *tbx6* activation
K_{FR}	0.3	threshold of Fgf/Erk for *tbx6* repression
K_{FA}	0.165	threshold of Fgf/Erk for *tbx6* activation
γ	0.42	production rate of Tbx6 protein by Fgf/Erk activation
η	2.0	degradation rate of Tbx6 protein by Ripply protein
F_0	0.75	Fgf/Erk signal intensity at the tailbud
x_f	100 μm	length scale of Fgf/Erk gradient
v_e	0.8	dpErk level at the tailbud
x_e	150 μm	boundary position of dpErk gradient
q	0.02 μm^{-1}	shape parameter of dpErk gradient
N	41	cell number
Δx	10 μm	cell size
u	2.0 $\mu m\ min^{-1}$	advection speed
x_h	$20\Delta x$	position where sensitivity of *her* to Tbx6 changes

Supplementary Table 2

List of primer pairs for the constraction of pGL3-r2-bamH-dN

MUT1-1	GGAATTCTGTCCTCTAGTGGAGGCATG	AACAGAAGATTTTTTCAGCTCTTACTGGACATCTG
MUT1-2	TAAGAGCTGAAAAAATCTTCTGTTTCTCCTTTCGA	GTTGTGGTTTGTCCAAACTCATC
MUT2-1	GGAATTCTGTCCTCTAGTGGAGGCATG	CTAACATGATTTTTTCCGATCTGTGGCGTGCGGGC
MUT2-2	ACAGATCGGAAAAAATCATGTTAGTGTTTCAGAGC	GTTGTGGTTTGTCCAAACTCATC
MUT3-1	GGAATTCTGTCCTCTAGTGGAGGCATG	CGGGTCTGATTTTTTCGATTGTGGAGGTGTGAGGT
MUT3-2	CCACAATCGCGGCTGTCAGACCCGAGGCGCGAAGT	GTTGTGGTTTGTCCAAACTCATC
MUT4-1	GGAATTCTGTCCTCTAGTGGAGGCATG	CGGAAAGCGTTTTTTACAAACGGTGACTTCGCGCC
MUT4-2	ACCGTTTGTAAAAAACGCTTTCCGGGTATTAAAGC	GTTGTGGTTTGTCCAAACTCATC
MUT5-1	GGAATTCTGTCCTCTAGTGGAGGCATG	AGAATGATGTTTTTTCTGAAGTCTGGAAGCTTTAA
MUT5-2	AGACTTCAGAAAAAACATCATTCTGATCGGAAACA	GTTGTGGTTTGTCCAAACTCATC
MUT67-1	CTGCAGGAATTCGATTTTCGCTGTGGTGACCCTGG	TAGGGTTCACTTTCGTCTCCATAGAAACGTGGT
MUT67-2	TATGGAGACGAAAGTGAACCCTAATCCACAACA	GACGAGGAGCCCTTTTCTTTTGAGGGACTGAGAGC
MUT67-3	CTCAAAAGAAAAGGGCTCCTCGTCCTCGTAATAT	ATCGATAAGCTTGATGAGGGAGAGATGGGCACTAG
MUT89-1	GCGGCCGCTCTAGAACTAGTGGATCCATGCTTTC	CTCCCATTTTTGAGTCTCTCACGCAGAGAAATCTC
MUT89-2	GAGAGACTCAAAAATGGGAGGTTAACCTGCTGCTG	CCGGAGGAAACCTTTGCGAACGCAGGGAGAACATG
MUT89-3	CCCTGCGTTCGCAAAGGTTTCCTCCGGGAGCTCCG	ATCGATAAGCTTGATTGAAACACTCGAGCTGCTGC
