# Supplementary material for: Ripply suppresses Tbx6 to induce dynamic-to-static conversion in somite segmentation
Source: Nat Commun. 2023 Apr 13;14:2115. doi: 10.1038/s41467-023-37745-w (PMC10102234; doi:10.1038/s41467-023-37745-w)
Supplement: Supplementary file 3 — Description of Additional Supplementary Files [file 41467_2023_37745_MOESM3_ESM.pdf]

Description of Additional Supplementary Files

File name: Supplementary Movie 1
Description: Time lapse movies of a wild-type embryo in which endogenous Her7 can be visualized by tagging with three copies of the Achilles, fast-maturing YFP variant at 1 frame/min.

File name: Supplementary Movie 2
Description: Time lapse movies of a *ripply1; ripply2* double-mutant embryo in which endogenous Her7 can be visualized by tagging with three copies of the Achilles, fast-maturing YFP variant at 1 frame/min.

File name: Supplementary Movie 3
Description: The result of mathematical simulation of wild-type embryos showing temporal changes in Her protein (green), dpErk (orange), Tbx6 protein (cyan), *ripply-intron* mRNA (magenta) and Ripply protein (black). The horizontal axis indicates space (μm: anterior on the left) and the vertical axis indicates relative levels of mRNA and proteins.

File name: Supplementary Movie 4
Description: The result of mathematical simulation without the positive feedback loop of Tbx6 protein. Spatial patterns of Her protein (green), *ripply-intron* mRNA (magenta), Ripply protein (black), Tbx6 protein (cyan) and dpErk protein (orange) are shown. The horizontal axis indicates space (μm: anterior on the left) and the vertical axis indicates relative levels of mRNA and proteins. The positive feedback loop in the equation for Tbx6 protein levels was replaced with a constant value.

File name: Supplementary Movie 5
Description: The result of mathematical simulation of *ripply*-defective embryos showing temporal changes in *her-intron* mRNA (green) and *her-exon* mRNA (magenta), compared with those of dpErk protein (orange) and Tbx6 protein (cyan). (Top) Simulation of wild type with *ripply* transcription. (Bottom) Simulation of *ripply* KO by setting the maximum transcription rate of *ripply* mRNA to zero. The horizontal axis indicates space (μm: anterior on the left) and the vertical axis indicates relative levels of mRNAs and proteins. To show oscillation in the anterior part, we used an array of $N = 63$ cells. The array represents the tissue region 630 μm anterior to the tailbud.

File name: Supplementary Movie 6
Description: The result of mathematical simulation of *her*-defective embryos showing temporal changes in *ripply-intron* mRNA (magenta) with those of dpErk (orange) and Tbx6 proteins (cyan). The horizontal axis indicates space (μm: anterior on the left) and the vertical axis indicates relative levels of mRNA and proteins. At $t = 82$ min, dpErk activity was reduced by 10-fold to model the effect of MEK1 inhibitor. To model *her* KO, we multiplied the transcription term of *her* mRNA by zero.

File name: Supplementary Movie 7
Description: The result of mathematical simulation of wild-type embryos, similar to Supplementary Movie 3, but with a stepwise retreat of dpErk activity. Spatial patterns of Her protein (green), *ripply-intron* mRNA (magenta), Ripply protein (black), Tbx6 protein (cyan) and dpErk protein (orange) are shown. The horizontal axis indicates space (μm: anterior on the left) and the vertical axis indicates relative levels of mRNA and proteins. The position of the anterior border of dpErk protein shifts at each oscillation cycle.

File name: Supplementary Software 1
Description: The C program and Mathematica codes that were used to simulate the mathematical model and visualize results.
