# Supplementary figures and images for: Ripply suppresses Tbx6 to induce dynamic-to-static conversion in somite segmentation
Source: Nat Commun. 2023 Apr 13;14:2115. doi: 10.1038/s41467-023-37745-w (PMC10102234; doi:10.1038/s41467-023-37745-w)

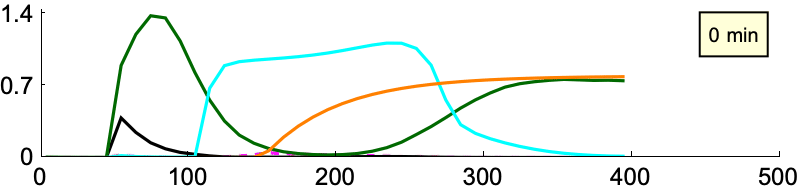

Supplement: Supplementary file 11 — Supplementary Software 1 [file 41467_2023_37745_MOESM11_ESM.zip › Supplementary_Software/WT_simulations/x_vs_ripi_Rip_Her_Tbx_Erk_lab_ref.gif]

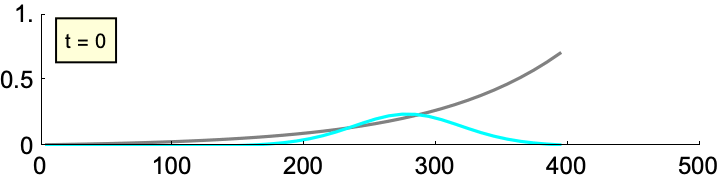

Supplement: Supplementary file 11 — Supplementary Software 1 [file 41467_2023_37745_MOESM11_ESM.zip › Supplementary_Software/WT_simulations/x_vs_Fgf_Tbxact_lab_ref.gif]
