# Supplementary material for: Ripply suppresses Tbx6 to induce dynamic-to-static conversion in somite segmentation
Source: Nat Commun. 2023 Apr 13;14:2115. doi: 10.1038/s41467-023-37745-w (PMC10102234; doi:10.1038/s41467-023-37745-w)
Supplement: Supplementary file 12 — Reporting Summary [file 41467_2023_37745_MOESM12_ESM.pdf]

nature portfolio

Corresponding author(s): Shinji Takada
Taijiro Yabe
Koichiro Uriu

Last updated by author(s): Mar 14, 2023

Reporting Summary

Nature Portfolio wishes to improve the reproducibility of the work that we publish. This form provides structure for consistency and transparency in reporting. For further information on Nature Portfolio policies, see our Editorial Policies and the Editorial Policy Checklist.

Statistics

For all statistical analyses, confirm that the following items are present in the figure legend, table legend, main text, or Methods section.

n/a	Confirmed	
☐	☒	The exact sample size (*n*) for each experimental group/condition, given as a discrete number and unit of measurement
☐	☒	A statement on whether measurements were taken from distinct samples or whether the same sample was measured repeatedly
☐	☒	The statistical test(s) used AND whether they are one- or two-sided<br>*Only common tests should be described solely by name; describe more complex techniques in the Methods section.*
☒	☐	A description of all covariates tested
☐	☒	A description of any assumptions or corrections, such as tests of normality and adjustment for multiple comparisons
☐	☒	A full description of the statistical parameters including central tendency (e.g. means) or other basic estimates (e.g. regression coefficient) AND variation (e.g. standard deviation) or associated estimates of uncertainty (e.g. confidence intervals)
☐	☒	For null hypothesis testing, the test statistic (e.g. *F*, *t*, *r*) with confidence intervals, effect sizes, degrees of freedom and *P* value noted<br>*Give P values as exact values whenever suitable.*
☒	☐	For Bayesian analysis, information on the choice of priors and Markov chain Monte Carlo settings
☒	☐	For hierarchical and complex designs, identification of the appropriate level for tests and full reporting of outcomes
☒	☐	Estimates of effect sizes (e.g. Cohen's *d*, Pearson's *r*), indicating how they were calculated

Our web collection on statistics for biologists contains articles on many of the points above.

Software and code

Policy information about availability of computer code

Data collection	Laica SP8 (LAS X; 3.1.5) and Zeiss Light-sheet nicrosoppe (Zen) were used for image data collection. TAL Effector Nucleotide Targeter 2.0 (https://tale-nt.cac.cornell.edu/node/add/talen-old) was used for the design of TALEN. Custom code written in C language which is attached in the "Supplementary Software" was used for numerical simulations of the mathematical model.
Data analysis	ImageJ(FIJI; 2.0.0) , R (4.1.2) and Genetyx-mac were used for image data analysis, statical analysis and DNA sequence analysis, respectively.

For manuscripts utilizing custom algorithms or software that are central to the research but not yet described in published literature, software must be made available to editors and reviewers. We strongly encourage code deposition in a community repository (e.g. GitHub). See the Nature Portfolio guidelines for submitting code & software for further information.

Data

Policy information about availability of data

All manuscripts must include a data availability statement. This statement should provide the following information, where applicable:

- Accession codes, unique identifiers, or web links for publicly available datasets
- A description of any restrictions on data availability
- For clinical datasets or third party data, please ensure that the statement adheres to our policy

Raw data associated the figures are included in the Source Data file. All the other data are available within the article and its Supplementary Information.

Human research participants

Policy information about studies involving human research participants and Sex and Gender in Research.

Reporting on sex and gender	not applicable' in all the below fields
Population characteristics	not applicable' in all the below fields
Recruitment	not applicable' in all the below fields
Ethics oversight	not applicable' in all the below fields

Note that full information on the approval of the study protocol must also be provided in the manuscript.

Field-specific reporting

Please select the one below that is the best fit for your research. If you are not sure, read the appropriate sections before making your selection.

☒ Life sciences ☐ Behavioural & social sciences ☐ Ecological, evolutionary & environmental sciences

For a reference copy of the document with all sections, see nature.com/documents/nr-reporting-summary-flat.pdf

Life sciences study design

All studies must disclose on these points even when the disclosure is negative.

Sample size	No sample size calculation was performed. Detailed n number are provided in the figures or figure legends. The sample size was determined according to previous studies using similar analyses (Shih et al. Development 2016, Haraoka et al.Nat. Commun.2022 and Ban et al.Development 2019)
Data exclusions	No data was excluded from the analysis in this study.
Replication	At least two independent experiments were taken to verify the reproducibility of the experimental findings in all experiments. All experiments were reliably reproduced.
Randomization	After the removal of unhealthy embryos before the onset of somitogenesis, embryos were randomly allocated to experimental groups. In the transplantation assay and transient HA-ripply1 expression assay in Fig.1, Fig.6 and Fig.S2, the embryos were sorted by expression of fluorescent signals of Rhordamin and eGFP respectively before the random allocation.
Blinding	No blinding was used because all experiments were conducted and analyzed by one person. Genotyping of each embryo was performed automatically, regardless of the results of experiments with each embryo.

Reporting for specific materials, systems and methods

We require information from authors about some types of materials, experimental systems and methods used in many studies. Here, indicate whether each material, system or method listed is relevant to your study. If you are not sure if a list item applies to your research, read the appropriate section before selecting a response.

Materials & experimental systems

n/a	Involved in the study	
☐	☒	Antibodies
☐	☒	Eukaryotic cell lines
☒	☐	Palaeontology and archaeology
☐	☒	Animals and other organisms
☒	☐	Clinical data
☒	☐	Dual use research of concern

Methods

n/a	Involved in the study	
☒	☐	ChIP-seq
☒	☐	Flow cytometry
☒	☐	MRI-based neuroimaging

Antibodies

Antibodies used	Rabbit anti-GFP Antibody 1:2000, Thermo Fisher; A-11122, lot no. 2015992<br>Mouse anti-dpERK Antibody 1:2000, Sigma; M9692, batch#0000102442<br>Mouse anti-V5 Antibody 1:2000, Invitrogen; 46-0705, lot no.505729<br>Mouse anti-Tbx6 Antibody 1:200 gifted from Dr. Oates-AC<br>Rabbit anti-Tbx6 Antibody 1:500 generated in Dr. Takada's lab.<br>Gaot HRP-labeled anti-mouse IgG Antibody 1:300 Promega; W402B, lot no. 39709501<br>Goat HRP-conjugated anti-Rabbit IgG Antibody 1:2000 Jackson Lab.111-035-114 74426<br>mouse Anti-γ-Tubulin antibody (GTU88)1:1000 Sigma; T6557, lot no. unknown<br>Goat anti-mouse IgG Alexa 647 1:800 ThermoFisher;A21235, lot no. 2031175<br>Goat anti-rabbit IgG Alexa 6471:800 ThermoFisher;A21245, lot no. 1037285<br>Rabbit anti-fibronectin antibody 1:200 Sigma; F3648, lot no. unknown<br>Goat anti-rabbit IgG Alexa 488 1:700 ThermoFisher; A11008, lot no. 1853312<br>Mouse rat anti-HA antibody (3F10) 1:500 Roche; 12158167001 lot no.11867423001<br>Chicken anti-GFP antibody 1:500 Abcam; ab13970, lot no. GR3361051-14<br>Goat anti-chicken IgG Alexa 488 1:700 ThermoFisher; A11039, lot no. 1869581<br>Goat anti-rat IgG Alexa 555 1:700 ThermoFisher; A21434, lot no. 1423054<br>Sheep Anti-Digoxigenin-AP, Fab fragments 1:10000 Roche; 11093274910, lot no.32871923<br>Sheep Anti-Digoxigenin-POD, Fab fragments 1:1000 Roche; 11207733910, lot no.43500600<br>Rabbit anti-b-catenin, 1:1000 Sigma; C2206, lot no. 099k4828<br>Goat anti-mouse IgG Alexa 488, 1/700; Thermo Fisher; A11001, lot no. unknown
Validation	Primary Antibodys used for IHC<br>Rabbit anti-Tbx6 antibody was validated in Wanglar et al., PLOS one, 2014 by Westerni blotting with Tbx6 expressing HEK293t cells.<br>mouse anti-Tbx6 antibody was validated in Winder et al., Development, 2015 by IHC using WT and tbx6 mutant embryo.<br>About commercially available antibodies the validation statements on the manufacturer's website are as follows.<br>Rabbit anti-GFP Antibody (#A-11122); https://www.thermofisher.com/antibody/product/GFP-Antibody-Polyclonal/A-11122<br>Mouse anti-dpERK Antibody (#M9692); https://www.sigmaaldrich.com/JP/en/product/mm/mabn842<br>Mouse anti-V5 Antibody (#46-0705); https://www.thermofisher.com/antibody/product/V5-Tag-Antibody-Monoclonal/R960-25<br>mouse Anti-γ-Tubulin antibody;GTU88 (#T6557); https://www.sigmaaldrich.com/JP/en/product/sigma/t6557<br>Rabbit anti-fibronectin antibody (#F3648); https://www.sigmaaldrich.com/JP/en/product/sigma/f3648<br>Mouse rat anti-HA antibody; 3F10 (#12158167001);https://www.sigmaaldrich.com/JP/en/product/roche/12158167001<br>Chicken anti-GFP antibody (#ab13970); https://www.abcam.com/products/primary-antibodies/gfp-antibody-ab13970.html<br>Rabbit anti-b-catenin (#C2206); https://www.sigmaaldrich.com/JP/en/product/sigma/c2206<br><br>Antibodys used for in situ hybridization analysis<br>Sheep Anti-Digoxigenin-AP, Fab fragments (#11093274910); https://www.sigmaaldrich.com/JP/en/product/roche/11093274910<br>Sheep Anti-Digoxigenin-POD, Fab fragments (#11207733910); https://www.sigmaaldrich.com/JP/en/product/roche/11207733910

Eukaryotic cell lines

Policy information about cell lines and Sex and Gender in Research

Cell line source(s)	HEK 293T were gifted by Dr. Takeichi (RIKEN)
Authentication	The cells were not authenticated.
Mycoplasma contamination	All cell lines were tested negative for mycoplasma contamination.
Commonly misidentified lines (See ICLAC register)	No cell lines listed were used in this study.

Animals and other research organisms

Policy information about studies involving animals; ARRIVE guidelines recommended for reporting animal research, and Sex and Gender in Research

Laboratory animals	Zebrafish with the TL2 background were used as the wild type. Genetic background of Tg(her1:her1-Venus) and ti1 are mix of TL2 and original background. Other mutant and transgenic fish carry mutations or transgenes in TL2 bacground. Eggs were collected by crossing of 4-12 month old parents fish to use experiments.
Wild animals	No wild animal was used in this study.
Reporting on sex	In zebrafish, sex is considered to be determined after the embryogenesis. Therefore, in this study all of experiment were performed before the sex determination of embryos.

Field-collected samples	No field collected sample was used in this study.
Ethics oversight	This study was performed in accordance with Guidelines for Animal Experimentation of the National Institutes of Natural Sciences, with approval of the Animal Care and Use Committee (IACAC) of the National Institutes of Natural Sciences.

Note that full information on the approval of the study protocol must also be provided in the manuscript.
